# Lemierre's syndrome due to community-acquired meticillin-resistant *Staphylococcus aureus *infection and presenting with orbital cellulitis: a case report

**DOI:** 10.1186/1752-1947-2-374

**Published:** 2008-12-08

**Authors:** Tamilarasu Kadhiravan, Paramasivan Piramanayagam, Amit Banga, Rajiva Gupta, Surendra K Sharma

**Affiliations:** 1 Department of Medicine, All India Institute of Medical Sciences, New Delhi, India

## Abstract

**Introduction:**

Lemierre's syndrome is septic thrombophlebitis of the internal jugular vein leading to metastatic septic complications following an oropharyngeal infection. It is usually caused by the anaerobe, *Fusobacterium necrophorum*. Of late, meticillin-resistant *Staphylococcus aureus *is increasingly being recognised as a cause of community-acquired skin and soft tissue infections. We report a rare case of Lemierre's syndrome caused by community-acquired meticillin-resistant *Staphylococcus aureus *infection.

**Case presentation:**

A previously healthy 16-year-old girl presented with fever of 13 days duration, painful swelling around the right eye and diplopia followed by the appearance of pulmonary infiltrates. Imaging studies confirmed the clinical suspicion of bilateral jugular venous thrombosis with septic pulmonary embolism. Meticillin-resistant *Staphylococcus aureus *was isolated on blood cultures. The hospital course was complicated by massive haemoptysis and pulmonary aspiration necessitating mechanical ventilation. The patient subsequently made a complete recovery.

**Conclusion:**

Lemierre's syndrome, although rare, is a potentially lethal but treatable complication of head and neck sepsis. Early clinical recognition of Lemierre's syndrome and appropriate antibiotic treatment can be life-saving. One should consider the possibility of community-acquired meticillin-resistant *Staphylococcus aureus *infection in patients with suspected Lemierre's syndrome.

## Introduction

Lemierre's syndrome is septic thrombophlebitis of the internal jugular vein, secondary to an oropharyngeal infective focus, resulting in metastatic septic complications [1]. In the pre-antibiotic era, Lemierre's syndrome was a relatively common condition with a high case-fatality rate. With the advent of antibiotics, it has become a rarity nowadays. However, over the past decade, a resurgence in the number of reported cases has been observed, attributable to judicious use of antibiotics in primary care [2]. Usually, it is caused by the anaerobic oral commensal, *Fusobacterium necrophorum *[1].

Meticillin-resistant *Staphylococcus aureus *(MRSA) is a prototypical nosocomial pathogen. However, over the past few years, it is being increasingly reported as an important cause of serious skin and soft tissue infections acquired in the community among individuals without any of the conventionally recognised risk factors for MRSA infection [3]. Lemierre's syndrome following community-acquired MRSA (CA-MRSA) infection is very rarely reported in the literature.

## Case presentation

A 16-year-old previously healthy girl presented with high-grade, intermittent fever of 13 days duration. The symptoms had started as an episode of sore throat and fever. A few days later, the patient developed pain and swelling around the right eye accompanied by diplopia. She also had dry cough of 1 day duration. There was no history of dyspnoea, neck pain, headache, vomiting, photophobia, or convulsions. On examination, the patient was febrile, alert, and was in no apparent respiratory distress. Oral cavity and oropharynx were unremarkable. A right periorbital swelling with minimal ipsilateral proptosis and dysconjugate gaze was evident. Movement of the right eye was restricted in all directions and was painful, suggesting orbital cellulitis. Visual acuity was 6/6 in both eyes. The right pupil was slightly dilated (6 mm); however, reaction to light was normal. Funduscopy revealed blurring of optic disc margins bilaterally.

On careful examination, prominent superficial veins were apparent over the neck (Figure not shown). Jugular venous waves were not discernible; instead, firm, cord-like structures were palpable bilaterally running alongside the sternomastoid muscles. Bronchial breath sounds and coarse crackles were present over the right lower lung field. Investigations revealed haemoglobin 7.0 g/dL, total leucocyte count 20,800 cells/μL, neutrophils 85%, lymphocytes 15%, and platelets 131,000/μL; blood chemistry was creatinine 0.8 mg/dL, bilirubin 0.7 mg/dL, albumin 2.4 g/dL, aspartate aminotransferase 32 IU/L, alanine aminotransferase 11 IU/L, and alkaline phosphatase 98 IU/L. A chest radiograph showed bilateral patchy air-space infiltrates. A diagnosis of Lemierre's syndrome was considered; after drawing blood cultures, intravenous co-amoxiclav and metronidazole were initiated.

**Figure 1:**
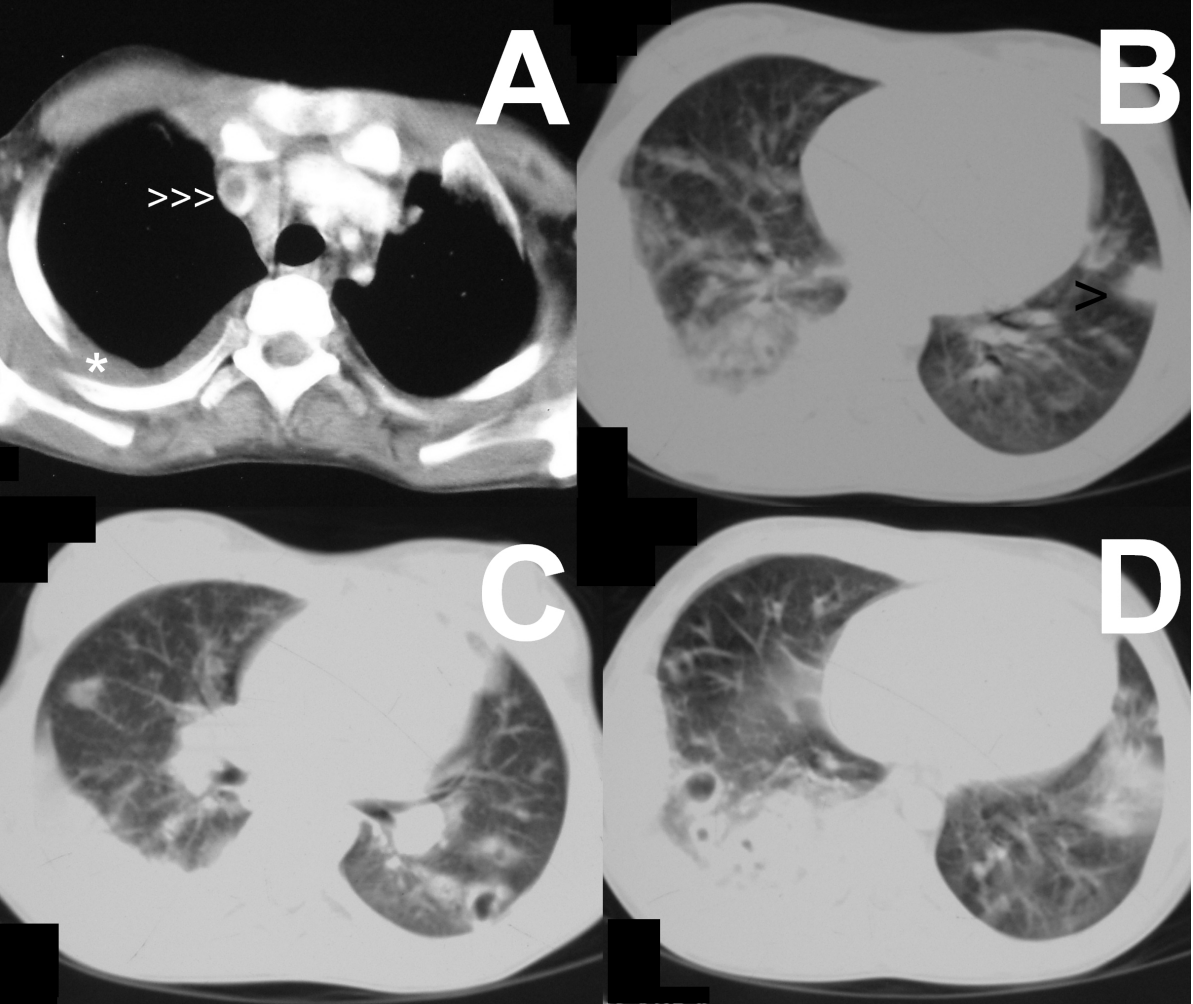
**Venous thrombosis and septic pulmonary embolism**. Contrast-enhanced computed tomographic images of chest showing an intraluminal filling defect caused by the thrombus in the superior vena cava (A, arrowheads), pleural-based wedge-shaped pulmonary infiltrate in left upper lobe (B, arrowhead), and cavitating infiltrates in bilateral lower lobes (C, D). Right-sided pleural effusion is also seen (A, asterisk).

**Figure 2:**
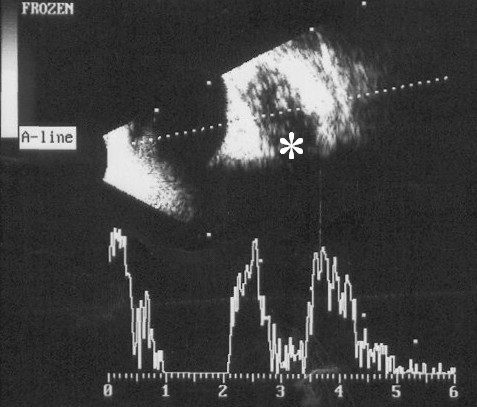
**Orbital cellulites**. High-resolution (10 MHz) ultrasonogram of right orbit showing an ill-demarcated hypoechoic collection (asterisk) with internal echoes, behind the globe. The scale bar at the bottom is in centimetres.

A contrast-enhanced computed tomogram (CECT) of head and neck showed an enhancing hypoattenuating collection superonasal to the right globe. The lumen of the right internal jugular vein was completely replaced by a thrombus, and the contralateral jugular vein also had incomplete obliteration by thrombus. On serial sections, the thrombus was found extending up to the level of the superior vena cava (Figure 1A). A CECT of chest and abdomen showed multiple bilateral peripherally placed wedge-shaped pulmonary infiltrates, some of them forming cavities (Figure 1B–D), suggestive of septic pulmonary embolism. No intra-abdominal abscess was noted. There was a moderate right-sided pleural effusion which was aspirated. The pleural fluid was clear looking and was exudative in nature; Gram's stain revealed no organisms, and cultures were sterile. An orbital ultrasonogram revealed a hypoechoic collection (16 × 9 mm) superonasal to the right globe (Figure 2). Magnetic resonance venography ruled out a cavernous sinus thrombosis. Antiphospholipid, antineutrophil cytoplasmic, and human immunodeficiency virus antibodies were negative. An echocardiography was not performed; however, no murmur or rub was apparent on careful clinical examination.

On the third hospital day, the patient had a bout of massive haemoptysis and pulmonary aspiration. She was intubated and mechanically ventilated. By this time, the blood cultures drawn at admission were growing meticillin-resistant *Staphylococcus aureus *(MRSA) susceptible to vancomycin, teicoplanin, and clindamycin; no anaerobe was isolated.

Subsequently, the antibiotics were changed to vancomycin, metronidazole, and cefoperazone-sulbactam. With continued antibiotic therapy and assisted ventilation, the patient made an uneventful recovery and was successfully extubated on day 7. On retrospective questioning, the patient denied a history of visit or admission to any health care facility or invasive medical procedure in the recent past. Repeat blood cultures were sterile; pulmonary infiltrates and pleural effusion showed resolution. The patient was discharged home on oral linezolid and metronidazole for 4 weeks. A month later, she was doing well without any functional limitations.

## Discussion

Most cases of Lemierre's syndrome occur in previously healthy persons aged 16 to 25 years [4]. Lemierre's syndrome is suspected clinically in less than 15% of cases, and isolation of *F. necrophorum *is the usual clue that points to the diagnosis retrospectively [4]. Apart from oropharyngeal infections, rarely mastoiditis or an infected tooth may result in Lemierre's syndrome [4]. Orbital cellulitis as the primary focus has not been reported earlier. Metastatic infection involves the lungs most commonly (about 80%), followed by large joints. Infrequently, hepatosplenic and soft tissue abscesses may occur. Other than *F. necrophorum*, organisms such as *Bacteroides *sp., *Eikinella *sp., *Streptococcus *sp., and *Staphylococcus epidermidis *have been reported to cause Lemierre's syndrome [4]. There are only two previous reports of Lemierre's syndrome caused by CA-MRSA infection [5,6].

MRSA, a prototypical nosocomial pathogen, is increasingly being recognised worldwide as a cause of community-acquired infections as well [3]. However, the CA-MRSA strains are inherently different from nosocomial MRSA strains and are probably more virulent than the latter. In the United States, 60% of patients presenting with skin and soft tissue infections have CA-MRSA infection [7]. Reliable data on the prevalence of CA-MRSA infection in developing countries are lacking. In two small studies from India, 1.4% and 11% of community-acquired pyoderma were found to be caused by MRSA [8,9].

Ability to induce aggregation of platelets is considered an important virulence factor of *F. necrophorum *that promotes the development of septic thrombophlebitis [2]. Gonzalez *et al. *had recently reported a case series of nine patients with CA-MRSA osteomyelitis and associated leg vein thrombosis; four of them had septic pulmonary embolism [10]. Like *F. necrophorum*, it seems, CA-MRSA also has a tendency to promote venous thrombosis. *F. necrophorum *elaborates an extracellular heat-stable leucocidin. Interestingly, analogous to this, CA-MRSA strains are also known to produce a leucocidin, the Panton-Valentine leucocidin [3]. Such a similarity of CA-MRSA with *F. necrophorum *could possibly explain the development of Lemierre's syndrome in our patient.

Timely initiation of appropriate antibiotics is the crux of management in Lemierre's syndrome. *F. necrophorum *is generally susceptible to clindamycin and metronidazole. Metronidazole monotherapy, however, is not recommended considering the possibility of polymicrobial infections. Metronidazole combined with high-dose penicillin or clindamycin as a single agent is recommended, to be administered for prolonged periods (3 to 6 weeks) [4]. Although clindamycin is active against CA-MRSA, it should not be used as the sole agent in severe CA-MRSA infections. Vancomycin is recommended in such cases; daptomycin, tigecycline, and quinupristin-dalfopristin are alternative drugs available. Drainage of pus collections may be needed if response to antibiotics is suboptimal. The role of anticoagulation in patients with Lemierre's syndrome is controversial.

## Conclusion

Physicians need to be aware of Lemierre's syndrome as a rare but potentially lethal complication of head and neck sepsis. Appropriate antibiotic therapy can be life-saving. With the emergence of CA-MRSA, one should keep in mind the possibility of CA-MRSA infection in patients with suspected Lemierre's syndrome.

## Consent

Written informed consent could not be obtained in this case since the patient is now lost to follow-up. We believe that this case report contains a worthwhile clinical lesson which could not be made as effectively in any other way. We expect that the patient and her family would not object to the publication since every effort has been made so that she remains anonymous.

## Competing interests

The authors declare that they have no competing interests.

## Authors' contributions

TK analysed and interpreted the patient data and drafted the manuscript. PP provided clinical care to the patient, performed the literature search, and was a major contributor in writing the manuscript. AB, RG, and SKS participated in the diagnostic work-up and clinical care of the patient, contributed significantly to the interpretation of the patient data, and revised the manuscript for important intellectual content. All authors read and approved the final manuscript.
